# The role of knowledge and fatalism in college students related to the earthquake-risk perception

**DOI:** 10.4102/jamba.v12i1.954

**Published:** 2020-09-30

**Authors:** Furqan I. Aksa, Sugeng Utaya, Syamsul Bachri, Budi Handoyo

**Affiliations:** 1 Department of Geography, Samudra University, Langsa, Aceh, Indonesia; 2 Department of Geography Education, State University of Malang, Malang, Indonesia; 3 Department of Geography, Faculty of Social Science, State University of Malang, Malang, Indonesia

**Keywords:** knowledge, fatalism, earthquake, risk perception, geography

## Abstract

At present, the earthquake-risk perception research in Aceh only focuses on the general public. Limited research examines earthquake-risk perceptions amongst students. This exploratory study is focused on geography education students because this study programme has integrated disaster education into its curriculum. This study aims to find the extent to which earthquake knowledge and fatalism beliefs affect earthquake-risk perception. The survey was conducted on 210 students using questionnaires. Using the Spearman correlation test, the results of this study revealed that there was a positive and significant relationship between earthquake knowledge and risk perception with coefficients (0.200) and significance (0.004). Meanwhile, fatalism beliefs have a negative and significant relationship to the perception of earthquake risk with correlation coefficient (−0.224) and significance (0.001). This means that the higher the fatalism attitude of students towards disasters, the lower the perception of earthquake risk. It is feared that fatalism will have an impact on the lack of disaster preparedness. Fatalism beliefs are complex issues that require joint efforts from governments, religious leaders, educational institutions and the media to reduce them.

## Introduction

Aceh region (located in Indonesia) is an earthquake-prone area. With regard to its geological location, Aceh is in a subduction zone. From 1990 to November 2004, Aceh had experienced more than 2200 earthquakes with powers above 6 in the Mw (Muksin et al. 2018). The largest earthquake occurred on 26 December 2004, with a magnitude of 9.15 Mw causing a tsunami disaster (Tsuji et al. 2006). The death toll consisted of 165 791 people, and 21 751 housing units and 169 education facilities were severely damaged (BNPB 2019). Recently, on 6 December 2016, an earthquake also occurred in Pidie Jaya District, Aceh, with a magnitude of 6.5 Mw, resulting in the death of 102 people (BNPB 2019).

The large number of casualties was caused by lack of knowledge, preparedness and poor building construction (Escaleras, Anbarci & Register 2007; Goda et al. 2018; Mertes & Dunne 2008). Disaster preparedness is strongly influenced by the perceptions of each individual (Rohrmann 2008). Disaster risk perception is influenced by four factors, namely, (1) risk factors, such as the likelihood and frequency of an event; (2) information factors, such as sources and levels of information; (3) personal factors, such as age, gender, profession and disaster experience and (4) contextual factors, such as family status, vulnerability index and area of residence (Wachinger et al. 2013). In addition, socio-economic, political and religious factors also play a role in influencing risk perception (Nakayama et al. 2019; Sasaki et al. 2019).

Assessing earthquake-risk perceptions is very important. Risk perception is very helpful in understanding and analysing human behaviour when they are exposed to disasters. People respond to earthquake according to their view of the hazard as perceptions and awareness of influence behaviour (Naseri & Kang 2017). However, after 15 years a large earthquake of 9.15 Mw struck Aceh, and there is still very limited research that assesses earthquake-risk perceptions amongst students. Most previous research only focused on earthquake-risk perception amongst the community.

Knowing the perception of earthquake risk amongst students is very important. Greer, Wu and Murphy (2018) mentioned that students are included in vulnerable groups when disasters occur. Most of the students are newcomers from various regions, so they do not have direct experience of disasters in the local area (Lovekamp & Tate 2008). In addition, previous research also stated that students often underestimate disaster risk and are very rarely involved in disaster preparedness activities (Lovekamp & Tate 2008). This condition makes them unprepared to face disaster.

This is corroborated by several previous research findings, including a study conducted at the University of South Florida that revealed 58% of students at the University was not prepared to face a hurricane (Collins et al. 2009). Likewise with students of the University of Waterloo, Canada, the findings of a study conducted by Tanner and Doberstein (2015) found that the majority of students at the University did not have a disaster emergency preparedness kit and had barriers that limited disaster preparedness (Tanner & Doberstein 2015). However, students often become neglected compared to other vulnerable groups in disaster risk reduction programmes (Collins et al. 2009). This study aims to find the extent to which earthquake knowledge and fatalism affect the perception of earthquake risk amongst geography students. Accurately, this study will test the following hypotheses:

- Hypothesis 1 (H1): There is a positive and significant relationship between earthquake knowledge and earthquake-risk perception.
- Hypothesis 2 (H2): There is a negative and significant relationship between fatalistic beliefs and earthquake-risk perception.

The study of the relationship between knowledge and fatalism-related earthquake-risk perception is important because disaster is the relationship between natural events, natural risk and physical and social vulnerability (Quarantelli 1978, 2005). In a social context, social scientists mentioned that risk perception is socially constructed. This means that personal risk perception is closely related to the social and cultural conditions (Adomah Bempah & Olav Øyhus 2017).

In Addition, Wachinger et al. (2013) point out that knowledge, direct experience, values and attitudes influence the risk perception. Risk perception is also influenced by socio-economic, educational and religious beliefs (Yari, Zarezadeh & Ostadtaghizadeh 2019). Risk perception also plays a major role in motivating someone to take action when disaster strikes, such as avoiding disaster, adapting or even risking (Wachinger et al. 2013). The risk perception model developed by Renn and Rohrmann (2000a) also shows that knowledge, cultural and social factors influence risk perception. He further stated that risk perception is a combination of hazard attributes and personal philosophy based on experience and beliefs that are embedded in the norms, values and culture of each community (Baytiyeh & Öcal 2016; Renn & Rohrmann 2000a; Slovic 2000).

## Theoretical framework

### Risk perception of earthquake

Risk perception is the subjective assessment of the probability of a specified type of accident happening and how concerned we are with the consequences (Baytiyeh & Naja 2015). Research on risk perception is very helpful in understanding and analysing student behaviour when they are exposed to disasters (Muzenda-Mudavanhu, Manyena & Collins 2016). Risk perception has two different paradigms: psychometric approach developed by psychologists and cultural theory approach developed by anthropologists and sociologists (Armaş & Avram 2008). Psychometric approach emphasises evaluation studies related to communication of disaster risk, gender, race and demographic factors. Cultural theory approach includes the study of social and cultural influences on perspectives (Ainuddin, Routray & Ainuddin 2014). Meanwhile, Renn and Rohrmann (2000a) developed a model for disaster risk perception that integrates psychological, social and cultural factors. The model emphasises that knowledge is influenced by cultural factors and influences the processing of heuristic information that underlies risk assessment (Renn & Rohrmann 2000b). This study uses a risk perspective model developed by Renn and Rohrmann (2000b).

### Earthquake knowledge

Knowledge is the most important aspect of disaster risk reduction. Comprehensive knowledge about disasters can help in making the right decisions when disasters occur. Referring to the theory of the protective action decision model (PADM) developed by Lindell and Perry (2004), knowledge plays a role in influencing people in the decision-making process when a disaster occurs. Meanwhile, according to Shaw et al. (2004), knowledge has several levels, starting from knowing, realising, deepening and making decisions. Knowledge is gained through direct experience and disaster education. Knowledge greatly influences the perception of disaster risk (Adiyoso & Kanegae 2013). Previous research conducted by Botzen, Aerts and Van Den Bergh (2009) on the role of knowledge of the causes of flooding on the perception of flood risk in the Netherlands revealed that people who have less knowledge of the causes of floods also have low risk perceptions of flood disasters.

### Fatalism

According to the cultural theory of risk based on Thompson et al. (1990), fatalism is an attitude that believes that everything that happens in the community environment is beyond their control. Fatalism is closely related to internal and external controls. According to Rotter (1966), an external monitor occurs when someone assumes that something that happens to him or her is not forever dependent on his or her actions but rather a fortune or fate. Meanwhile, internal control is a condition when individuals feel that an event depends on their behaviour (Rotter 1966). This opinion was corroborated by Rashwan and Jenkins (2017), which states that fatalism is a belief that significant events in life are outside of individuals. Those who hold to the views of fatalism believe in the power of nature or luck.

Confidence in external factors makes a person less likely to care about risk (Xue et al. 2014). For example, people who live in volcanic locations generally believe in external forces such as nature that will not do them any harm. The findings of a previous study conducted by Mei et al. (2013) revealed that trust in life and ‘Mbah Marijan’ (a single figure who is considered a guardian of Mount Merapi) made many people refuse to evacuate during the Merapi volcano eruption in Central Java in the year 2010 (Mei et al. 2013). The same case also happened to people who live in the mountains reaching Bromo in East Java. Research conducted by Bachri et al. (2015) revealed that people who live in the Bromo volcano region have the belief that if they do wrong to Bromo or have negative thoughts, Bromo will provide the same situation as thought (Bachri et al. 2015). Confidence in external control will make people fatalistic. The belief in fatalism will have an impact on the lack of risk perception and disaster preparedness (Baytiyeh & Naja 2016).

## Method

Data were collected from geography education students at two prominent universities in Aceh, namely, Syiah Kuala University and Samudra University. The geography education department was chosen because it is a study programme that has integrated disaster education into the higher education curriculum. This is in accordance with the mandate of the Lucerne Declaration on Geographical Education for Sustainable Development (Haubrich 2007), which emphasises the importance of the theme of disaster risk reduction and climate change integrated into the teaching of geography throughout the world.

In addition, the two universities were chosen because they are located in earthquake-prone areas. The Province of Aceh was the region of Indonesia that was most severely affected by the earthquake on 26 December 2004. Researchers coordinated with the heads of departments at the two universities to distribute questionnaires to students. The survey was conducted using online survey software. Researchers distributed questionnaire links to geography education students at the two universities. All students enrolled in the geography education study programme are required to participate in this research. At the time of data collection, the total number of geography education students at the two universities was 557. We received 210 complete surveys consisting of 101 from Syiah Kuala University and 109 from Samudra University. We did not offer additional credit because respondents who completed the survey were representative of 37.5% of the total population of geography students; 37.5% is considered a relatively high response rate amongst college students (Fosnacht et al. 2017).

### Measurement and instrumentation

The questionnaire to measure the level of knowledge, risk perception and fatalism beliefs contains 15 question items consisting of 5 question items about earthquake knowledge, 5 statement items related to earthquake-risk perception and 5 statement items fatalism beliefs (Table 1). Knowledge tests use the Guttman scale (true/false). True earns a score of 1, whilst wrong earns a score of 0. Earthquake-risk perception is measured by two variables, namely, perceived hazard probability and consequences. Risk perceptions and fatalistic beliefs are made in the form of a Likert scale (1–5). The questions in fatalism belief are the result of the development and modification of previous studies conducted by Baytiyeh and Öcal (2016) and Yari et al. (2019).

**TABLE 1:** Questionnaire for knowledge, fatalism beliefs and risk perception.

Questionnaire determinants	Questions	Mean	SD
**Knowledge** Means (0.83)	1. Can earthquakes be accurately predicted at the hour, day and place? Yes No	0.63	0.48
	2. Can the earthquake period be predicted? Yes No	1.00	0.00
	3. Is liquefaction one of the direct impacts that can occur after an earthquake? Yes No	0.75	0.44
	4. If an earthquake occurs whilst you are in a classroom, what do you do? Run to an open field (gathering point) Wait for evacuation orders Contact the authorities immediately	0.90	0.89
	5. If you feel a strong earthquake (lasting more than 20 s) and you are at the beach. What will you do? Run towards the evacuation route Wait for evacuation orders from the authorities Watch what happens	0.89	0.31
**FB** Means (3.75)	6. Earthquakes are signs from God	4.20	0.40
	7. Earthquakes are God’s way of testing our faith	3.86	0.42
	8. Earthquakes are punishments from God for the sins of some people	4.06	0.53
	9. God is likely to provide some assistance to me during an earthquake	2.80	0.40
	10. No action can be taken to reduce the impact of damage and destruction from an earthquake.	3.87	0.34
**RP** Means (3.59)	11. Large-scale earthquakes are likely to occur in Aceh in the future.	3.64	0.48
	12. There are several active seismic faults in Aceh.	3.66	0.47
	13. Earthquakes in neighbouring provinces can have an impact on Aceh.	3.70	0.46
	14. Tens of thousands of people might be injured or die if an earthquake occurs in Aceh.	3.63	0.48
	15. Buildings that are not designed to withstand earthquakes are likely to collapse when an earthquake occurs.	3.36	0.48

FB, fatalism belief; RP, risk perception.

### Data analysis

The research instruments were analysed using validity and reliability tests. Validity test aims to measure the extent to which the research instrument can be used to measure a variable. Measurement of normality is performed using the Shapiro–Wilk method. After that, a reliability test is conducted, which aims to measure the reliability of the research instruments. The instrument can be said to be reliable if it has a reliability coefficient of 0.6 or more. Cronbach’s alpha was used as the reliability test. After the research instrument proves valid and safe, it is followed by the Spearman correlation test. Spearman correlation is used because the scale of the data is ordinal, so the non-parametric inferential analysis is used (Table 4) (Myers & Sirois 2014). Data processing is achieved using SPSS 24 software.

### Ethical considerations

The authors declare that ethical clearance was not needed for the study.

## Result

Respondents were dominated by women (70.3%). The results of normality measurements show the value of the Shapiro–Wilk coefficient of 0.000 (Table 2). From these figures, it can be concluded that the data are normally distributed. The reliability measurement results show that the Cronbach’s alpha coefficient value is 0.649. This indicates that the data from the variables are classified as reliable to use.

**TABLE 2:** Test results of reliability and normality.

Reliability of statistics	Kolmogorov–Smirnova†			Shapiro–Wilk		
	Statistics	Df	Sig.	Statistics	Df	Sig.
Total	0.129	210	0.000	0.963	210	0.000

Cronbach’s alpha = 0.649, based on standardised items 584.

† , Cronbach’s alpha = 0.649, based on standardised items 584.

Meanwhile, Spearman correlation test results (Table 3) indicate a positive and significant relationship between knowledge earthquake-risk perception (coefficient = 0.200 and significance = 0.004 < 0:01). This finding is consistent with H1 and H2, indicating a negative and significant relationship between fatalism beliefs and earthquake-risk perception (correlation coefficient = −0.224 and significance = 0.001 (two tailed) < 0.01).

**TABLE 3:** Means and standard deviation.

Components	Means
Knowledge	0.83
Confidence fatalism	3.75
Risk perception	3.59

**TABLE 4:** Spearman correlation test results.

Variable		Perception	Knowledge	Fatalism
Perception	Correlation Coefficient	1000	0.200*	−0.224*
	Sig. (two tailed)	-	0.004	0.001
	*N*	210	210	210

* , Correlation is significant at the 0.01 level (two tailed).

## Discussion

The students’ average score of knowledge on earthquakes is relatively high. The five items of knowledge of earthquake questions have a mean of 0.83 on a 1-point scale. The high knowledge of earthquakes because of disaster education has been integrated into the geography education curriculum at Syiah Kuala University and Samudra University. Through geology, geomorphology and disaster geography, students were taught about earthquakes.

The high knowledge of earthquakes has a positive impact on risk perception (significance = 0.004). It supports the opinion expressed by Yari et al. (2019) that knowledge has a positive relationship with earthquake-risk perception. Most respondents agreed with the statement that a large-scale earthquake is likely to occur in Aceh in the future, with an average value of 3.64 on a 5-point scale. Besides, Acehnese students also believed that the earthquake would have major consequences on the people of Aceh. Most respondents believe that tens of thousands of people might be injured or die if an earthquake occurs in Aceh (means 3.63 on a 5-point scale).

The findings of this study are consistent with the cognitive learning theory developed by Ormrod (2008) that an information trained and practiced through elaboration will be stored in human memory forever. Furthermore, the information can be withdrawn to respond to a reaction (Ormrod 2008). Disaster education taught since 2007 continues to be believed to increase earthquake knowledge and is stored in the brains of students, so that they provide responses related to the perception of earthquake risk. In addition, the findings of this study use research on the relationship of knowledge with disaster risk perception conducted by Botzen et al. (2009) who found that people who had little knowledge of flooding had lower risk perceptions compared to those who knew about flooding.

The findings of this study provide empirical evidence that it is important to strengthen the knowledge of future earthquakes. Knowledge of earthquakes is believed to influence people in making decisions at the time of a disaster. According to the theory of PADM, knowledge is one of the most influential components in retrieval decisions when a disaster occurs (Lindell & Perry 2004).

Other findings from this study revealed that although students had high knowledge of earthquakes, they also held to the beliefs of fatalism. Statistical test results show that the mean fatalism value of Acehnese students is high (3.75 on a scale of 5). The average value of the statement ‘no action can be taken to reduce the impact of damage and destruction from an earthquake’ (3.87 on a scale of 5). This number indicates that most respondents agreed that there were not many ways to reduce the impact of the earthquake disaster. The findings of this study reinforce the cultural theory of risk developed by Thompson et al. (1990) that fatalism is an attitude believing that everything that happens in the community environment is beyond their control. In addition, this finding is consistent with the results of previous research conducted by Baytiyeh and Öcal (2016) who state that the high knowledge of earthquakes amongst Lebanese and Turkish students does not make them free from fatalism beliefs (Baytiyeh & Öcal 2016).

The high fatalism attitude of Acehnese students was allegedly because of the influence of the message conveyed by religious leaders. After the 2004 Indian Ocean earthquake and tsunami, most religious leaders in Aceh interpreted the disaster as a form of punishment from God for human sins. The message conveyed by religious leaders allegedly influenced the perception of earthquake risk amongst students. The findings of this study proved the risk perception model developed by Renn and Rohrmann (2000b), which states that risk perception is a combination of hazard attributes and personal philosophy based on experience and belief in the norms, values and culture of the local community (Renn & Rohrmann 2000b). Confidence in the values and norms in Acehnese society is thought to have influenced the perception of earthquake risk amongst students.

The high belief in the fatalism of Acehnese students has a negative relationship with risk perception (correlation value −0.224 and significance 0.001). The data explained that the higher attitude of the fatalism of students towards disasters resulted in lower perception of earthquake risk. This finding corroborates several studies in the field of psychology, which show that empirical fatalism beliefs have a negative effect on decision-making and human behaviour (Rashwan & Jenkins 2017). It is feared that fatalism will have an impact on the lack of disaster preparedness (Baytiyeh & Naja 2016; McClure, Allen & Walkey 2001). This is confirmed by the findings of previous research conducted by Baytiyeh and Naja (2016) which show that there is a negative relationship between fatalism beliefs and disaster preparedness amongst students in Lebanon.

Therefore, joint efforts are needed in reducing fatalism beliefs. The government, religious leaders and the media are expected to play a role in reducing fatalism beliefs. The role of religious leaders is very important to reduce fatalism beliefs. According to Ghafory-Ashtiany (2014), fatalism beliefs, especially in Islamic countries, are thought to be caused by misunderstanding of religious teachings. Religious leaders can play a role in providing a comprehensive understanding of disasters, so that people do not act fatalistic.

In addition, the media also plays a role in reducing fatalism beliefs. The way the media presents disaster damage information will affect public perception. For example, the information conveyed that damage from an earthquake cannot be prevented but will increase fatalism beliefs amongst the people (McClure et al. 2001). Therefore, the media needs to provide educational information such as efforts to prevent damage from earthquakes to reduce fatalism beliefs.

## Conclusion

The main objective of this study is to assess the relationship between knowledge and fatalism beliefs on earthquake perceptions amongst students. The results showed that knowledge has a positive and significant relationship affecting the perception of earthquake risk. The higher knowledge of earthquakes significantly increases the risk perception. This finding provides empirical evidence regarding the importance of increasing knowledge about disaster risk reduction. Comprehensive knowledge about disasters will increase risk perception. In the end, it will help in making the right decision when a disaster occurs.

Other findings of this study indicate that geography students have a high belief in fatalism during disasters. The high belief in fatalism has a negative relationship with the perception of earthquake risk. The higher belief in fatalism will cause low perception of earthquake risk. Therefore, this study suggests the need for joint efforts from the government, religious leaders and the media to reduce fatalism beliefs amongst students.
